# Phenotypic, Morphological and Adhesive Differences of Human Hematopoietic Progenitor Cells Cultured on Murine versus Human Mesenchymal Stromal Cells

**DOI:** 10.1038/srep15680

**Published:** 2015-10-26

**Authors:** Doreen Reichert, Jens Friedrichs, Steffi Ritter, Theresa Käubler, Carsten Werner, Martin Bornhäuser, Denis Corbeil

**Affiliations:** 1 Tissue Engineering Laboratories (BIOTEC), Technische Universität Dresden, 01307 Dresden, Germany; 2 Institute for Biofunctional Polymer Materials, Leibniz Institute of Polymer Research Dresden, 01069 Dresden, Germany; 3 Medical Clinic and Polyclinic I, University Hospital Carl Gustav Carus, 01307 Dresden, Germany; 4 DFG Research Center and Cluster of Excellence for Regenerative Therapies Dresden 01307 Dresden, Germany

## Abstract

Xenogenic transplantation models have been developed to study human hematopoiesis in immunocompromised murine recipients. They still have limitations and therefore it is important to delineate all players within the bone marrow that could account for species-specific differences. Here, we evaluated the proliferative capacity, morphological and physical characteristics of human CD34^+^ hematopoietic stem and progenitor cells (HSPCs) after co-culture on murine or human bone marrow-derived mesenchymal stromal cells (MSCs). After seven days, human CD34^+^CD133^–^ HSPCs expanded to similar extents on both feeder layers while cellular subsets comprising primitive CD34^+^CD133^+^ and CD133^+^CD34^–^ phenotypes are reduced fivefold on murine MSCs. The number of migrating HSPCs was also reduced on murine cells suggesting that MSC adhesion influences cellular polarization of HSPC. We used atomic force microscopy-based single-cell force spectroscopy to quantify their adhesive interactions. We found threefold higher detachment forces of human HSPCs from murine MSCs compared to human ones. This difference is related to the N-cadherin expression level on murine MSCs since its knockdown abolished their differential adhesion properties with human HSPCs. Our observations highlight phenotypic, morphological and adhesive differences of human HSPCs when cultured on murine or human MSCs, which raise some caution in data interpretation when xenogenic transplantation models are used.

Deciphering the cellular and molecular players involved in the homeostasis of the bone marrow (BM) niches is essential to gain insight into processes of hematopoietic stem and progenitor cell (HSPC) mobilization and homing to improve treatment options for patients with several hematological diseases. The use of various immunodeficient murine models and new imaging techniques, such as two-photon laser-scanning intravital microscopy, has increased our knowledge of mechanisms underlying the egress of HSPCs from, and their homing and lodging into the BM upon mobilization and transplantation, respectively^1^,^2^. The establishment of co-culture systems based on feeder cell layers (primary cells or cell lines) of human (h) origin has contributed to our understanding of hHSPC biology^3^,^4^,^5^. Using this approach, cell surface molecules involved in the adhesion of HSPCs to BM cellular constituents (e.g., multipotent mesenchymal stromal cells (MSCs)) and extracellular matrix components as well as secreted factors implicated in their crosstalk were identified^6^,^7^,^8^. Although more faithful models of the BM microenvironments are emerging^9^, HSPC niches still remain incompletely understood and their complexity is ever growing.

Recently, concerns about the use of animal models to study human cell biology became evident. Although humanized mice are extremely useful, transplanted hHSPCs often cannot fully reconstitute the blood system suggesting that certain factors produced by the human BM microenvironments are missing in the murine system^10^,^11^,^12^,^13^. Alternatively, the interactions between hHSPCs with surrounding cells and/or matrix molecules, and the binding of growth factors, which are essential for their proliferation and survival, might differ between species.

To investigate these issues, we set out to compare the behavior of hHSPCs growing on murine (m) *versus* hMSCs as feeder cell layers. MSCs were demonstrated to be an essential component of HSPC niches^14^. Others and we have established co-culture systems where mobilized peripheral blood CD34^+^ hHSPCs are grown on BM-derived hMSCs in a cytokine-driven protocol^4^,^15^. Using such systems, we could evaluate i) the expansion of hHSPCs; ii) their polarization and migration, and iii) the intercellular communication^4^,^6^,^16^,^17^. Here, we extended our experimental settings to MSCs isolated from murine BM^18^. By applying flow cytometry, time-lapse video and scanning electron microscopy (SEM) we found subtle differences in hHSPC expansion, phenotypic profiles, and polarization upon contact with mMSCs by comparison to human ones. These variations prompted us to quantitatively compare hHSPC adhesion strength on MSCs by atomic force microscopy (AFM)-based single-cell force spectroscopy (SCFS). We observed that detachment forces of hHSPCs are higher on mMSCs suggesting a difference in intercellular adhesion. We found that N-cadherin expressed by MSCs is the main cause for the differential adhesion force. This is in agreement with earlier reports highlighting the importance of this adhesion protein in the molecular crosstalk within the BM niche^19^,^20^. The divergence of the human cell-mouse cell interactions could partly explain why hHSPCs hosted in the murine BM microenvironment did not differentiate into all functional blood cells^13^. Therefore, our data raise some caution as to the interpretation of experimental results when murine models are used to study the primitive properties of human stem and progenitor cells.

## Results

### Characterization of murine mesenchymal stromal cells

Mouse MSCs were isolated by crushing femur and tibia followed by a collagenase treatment^18^. The plastic-adherent cells displayed spindle-shaped morphologies (Supplementary Fig. S1a). Flow cytometry and immunofluorescence microscopy showed that cells were positive for nestin, vimentin, CD29, CD44, CD71, CD105, CD140a, CD140b, CD146, CD166, CD325 (N-cadherin) and Sca-1 (Supplementary Fig. S2), and negative for Ter-119, CD11b, CD34, CD45, CD90.1, CD117 (stem cell factor receptor/c-kit), CD133 (prominin-1), CD135 (FMS-like receptor tyrosine kinase-3) and CD150 (Supplementary Fig. S3) as previously reported^18^. Recently, we described novel cell surface markers of BM-derived hMSCs^21^. We found CD97, CD239 and CD316, which were also expressed by mMSCs (Supplementary Fig. S2). However, they were negative for CD276 in contrast to hMSCs (Supplementary Fig. S3)^21^,^22^. CD90.2 and CD239 showed a heterogonous expression (Supplementary Fig. S2) and CD90.2 expression varied between mMSC preparations in agreement with Morikawa and colleagues^18^. The phenotypic signature of mMSCs was conserved up to 20 passages.

In addition, we investigated the general glycosylation pattern of mMSCs by probing them with 20 different lectins. Twelve lectins showed a positive reaction with mMSC (Supplementary Table S1). Interestingly, two of them display a differential binding between m and hMSCs; GSL I bindings being present in murine cells but absent in hMSCs, whereas PNA binding was observed only in hMSCs (Supplementary Fig. S4)^23^. The implication of such differential glycosylation remains to be determined. Nonetheless, it could influence the adhesion of MSCs to cellular components and surrounding matrix within BM compartments.

Given that certain gangliosides were proposed as potential markers of BM-derived hMSCs^24^, we probed GM1, GM3 and GD2 expression in mMSCs. Similar to hMSCs^23^, murine cells expressed both GM1 and GM3 (Supplementary Fig. S5). However, we failed to detect GD2 using mouse monoclonal 14.G2a. This is in line with a recent study questioning the 14.G2a immunoreactivity observed in hMSCs since GD2 could not be recognized by a chemical analysis suggesting a case of molecular mimicry^25^.

Besides phenotypic characterization, we evaluated the proliferation and differentiation capacities of primary mMSCs. The cells maintained their proliferative and clonogenic capacities as shown by CFU-F assays (Supplementary Fig. S1b). Their multipotential differentiation properties were conserved as demonstrated by Oil red O and Alizarin Red S labeling, after their culture in adipogenic or osteogenic differentiation media, respectively (Supplementary Fig. S1c). These properties were confirmed for passages 4, 10 and 20.

### *Ex vivo* expansion of human hematopoietic stem and progenitor cells cultured on murine or human mesenchymal stromal cells

**Figure 1:**
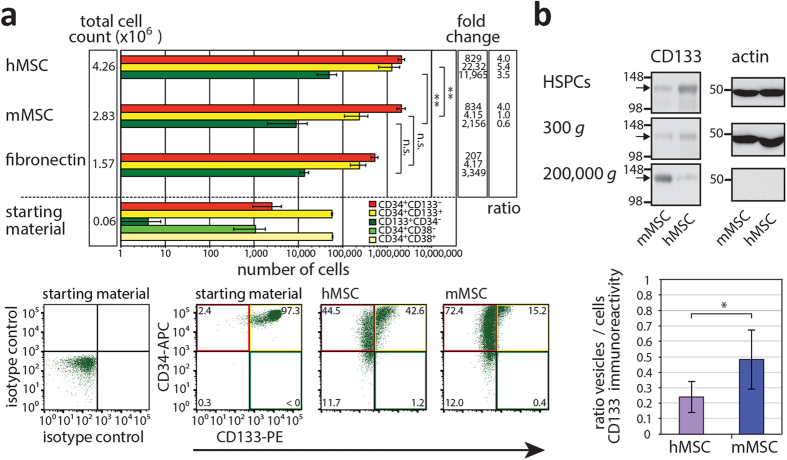
Expansion of human HSPCs cultured on murine or human MSCs and release of CD133^+^ membrane vesicles. (**a**) CD34^+^ HSPCs (6 × 10^4^ cells) isolated from mobilized peripheral blood (starting material) were cultured for one week either on murine (m) or human (h) MSCs or fibronectin prior to their analysis by flow cytometry for CD34 and CD38 or CD133. Total cell count, number of cells harboring a given phenotype (as indicated) and their expansion are shown. Fold change between cultivation with m or hMSCs or without versus starting material is indicated. Ratio of HSPC expansion with and without feeder cell layer is shown. Mean and standard deviation from four independent experiments using four distinct donors of HSPCs and two m or hMSCs preparations are shown. Note the abscissa is at a logarithmic scale (top panel). Representative dot plots are displayed (bottom panels). In the starting material, most of hHSPCs are CD34^+^CD133^+^ while CD133^+^CD34^–^ ones are very rare consistent with CD34 selection. The colored quadrants correspond to the cell phenotypes indicated in the upper panel. (**b**) After 7 days of HSPC–MSC co-culture, conditioned media were subjected to differential centrifugation for 5 min at 300 × *g*, 20 min at 1,200 × *g*, 30 min at 10,000 × *g* and 60 min at 200,000 × *g*. Adherent hematopoietic cells (HSPCs) were harvested, and centrifuged 5 min at 300 × *g.* All pellets were analyzed by immunoblotting for CD133 (arrow) and actin. Note that only half of the material was loaded for the HSPC fraction, and 1,200 and 10,000 × *g* fractions are not shown since no CD133 was detected therein. A representative experiment is displayed. The amount of CD133-immunoreactive material in each fraction was quantified, and ratios of vesicles (200,000 × *g*)/cells (HSPCs + 300 × *g*) are presented (mean ± standard deviation). Four independent experiments using distinct donors of HSPCs and MSC preparations were performed. Asterisks indicate a significant difference as calculated by two-tailed paired student’s t-test (*p < 0.05; **p < 0.01). N.s., not significant (*p* ≥ 0.05).

To evaluate the potential of mMSCs in supporting the expansion of hHSPCs, both cell types were co-cultured. Human HSPCs were purified from mobilized peripheral blood using a CD34^+^ MACS cell separation kit. They were 99% positive for CD34 with 98.20 ± 1.22% of them being CD34^+^CD38^+^ (Fig. 1a). In our starting materials, 95.75 ± 2.69% and 4.25 ± 2.69% of cells harbored CD34^+^CD133^+^ and CD34^+^CD133^–^ phenotype, respectively (Fig. 1a). The *ex vivo* expansion of the CD34^+^ HSPCs was implemented for 7 days in the presence of early-acting cytokines. They were cultivated either on confluent m or hMSCs, or on fibronectin-coated plates^4^. An optimal expansion of hematopoietic cells was only achieved in the presence of feeder cells (Fig. 1a, total cell count). The mean-fold expansion of nucleated cells was 47 and 71 in the presence of m and hMSCs, respectively, whereas only 26-fold in the absence of feeder cell layer (Fig. 1a, fibronectin). Neither fibronectin nor murine cells seemed to induce a significant hematopoietic cell death in comparison to hMSCs (data not shown). The phenotypic profile of hHSPCs was then extended to the CD133 antigen. Human CD133 is expressed on a subpopulation of CD34^+^ HSPCs, and a rare population of CD133^+^CD34^–^ HSPCs with primitive stem cell properties has also been described^4^,^26^,^27^,^28^. Immunoisolation based on CD133 is clinically used as an alternative to CD34^29^. Flow cytometry analyses revealed that mature CD34^+^CD133^–^ cells expand 4 times more on MSCs in comparison to fibronectin (Fig. 1a, ratio) in agreement with a previous study^4^. Surprisingly, those harboring CD34^+^CD133^+^ or CD133^+^CD34^–^ phenotype are poorly expanded on mMSCs (Fig. 1a). The numbers of CD34^+^CD133^+^ and CD133^+^CD34^–^ hHSPCs increased 22 and 12,000-fold on hMSCs, respectively, but only 4 and 2,000-fold on mMSCs. The latter data are comparable to those obtained in the absence of feeder cells (Fig. 1a, fold change). In conclusion, CD34^+^ hHSPCs can be expanded on both m and hMSCs, but those with more primitive phenotypes are less supported on mMSCs suggesting that the interaction of hHSPCs with hMSCs is more beneficial for the maintenance of hematopoietic stem cell properties.

### Release of CD133^+^ membrane vesicles by human hematopoietic stem and progenitor cells

Because the immunophenotypic signature alone might not necessarily correlate with the differentiation status of the cells, we investigated whether the reduced expansion of CD34^+^CD133^+^ and CD133^+^CD34^–^ hHSPCs on mMSCs might be explained by an early release of extracellular membrane vesicles containing CD133, which was demonstrated to occur during the differentiation process of HSPCs^6^. To investigate this cell biological issue, 7-day-old conditioned HSPC–MSC medium was collected and subjected to differential centrifugation followed by immunoblotting of the recovered pellets for CD133 (Fig. 1b). Interestingly, we observed a significant release of CD133^+^ membrane vesicles into the medium when CD34^+^ hHSPCs were cultured on mMSCs suggesting that the HSPC differentiation process is initiated earlier then on hMSCs (Fig. 1b, fraction 200,000 × *g*). It has to be noted that neither m nor hMSCs expressed CD133 (Supplementary Fig. S3, Ref. 4), and consequently the CD133^+^ membrane vesicles are derived exclusively from hHSPCs.

### Polarization and migration of human hematopoietic stem and progenitor cells cultured on murine or human mesenchymal stromal cells

**Figure 2:**
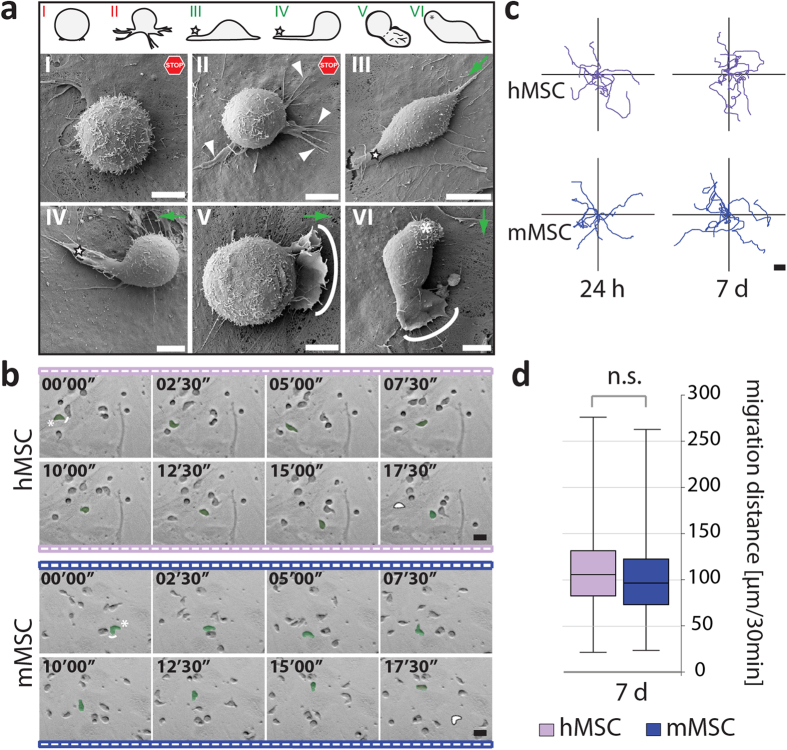
Polarization and migration of human HSPCs growing on murine or human MSCs. (**a**) Human (h) HSPCs growing on murine (m) MSCs for 7 days were analyzed by SEM. Six cellular shapes are depicted (top panel, models I-VI) with the corresponding photographs (middle and bottom panels). Adherent hematopoietic cells were either round with microvilli on their surface (I) or displayed large plasma membrane protrusions such as filopodium (II, arrowhead), magnupodium (III-IV, star) and lamellipodium (V, solid line). They could also develop a uropod at the rear pole (VI, asterisk). Models I-II show non-migrating cells (stop) whereas models III-VI exemplify migrating ones. Green arrows represent the direction of migration. (**b**) Individual frames taken from a time-lapse video show a hHSPC (green) moving on the surface of h or mMSCs after 24 hours of co-culture. Asterisk and white line indicate the rear and front pole of a migrating hHSPC, respectively (first frame). White spot marks its starting point (last frame). The elapsed time is shown in the upper-left corner. (**c**) Tracking diagrams depict the movement of 10 cells for 30 min. They were tracked after 24 hours and 7 days of co-culture on MSCs. (**d**) Box-and-whisker plots show the migration distance within 30 min of cells cultured on MSCs for 7 days. The boxes represent data from 25th–75th percentiles and 100% within the whiskers. Horizontal lines within the box represent median values. 100 cells were counted per condition (*n* = 3; CD34^+^ HSPCs were isolated from distinct donors and cultured on two different MSC preparations). No significant (n.s.) difference was observed as calculated by two-tailed paired student’s t-test (*p* ≥ 0.05). Scale bars: 5 μm (**a**), 20 μm (**b,c**).

Human HSPCs co-cultured on hMSCs adopt different polarized morphologies^4^,^16^,^29^,^30^. A SEM analysis revealed a similar situation when they are cultured on murine cells (Fig. 2a). Non-migrating hematopoietic cells displayed a spherical shape and developed numerous microvillus-like projections on their surface (Fig. 2a, model I). Thin filopodia that extended in various directions were also observed (model II, arrowheads). In contrast, migrating cells showed an elongated morphology with the formation of a magnupodium (models III, IV, star) or lamellipodium (models V, VI, solid line) at their front pole. A uropod could be observed at the rear pole (model VI, asterisk).

**Figure 3:**
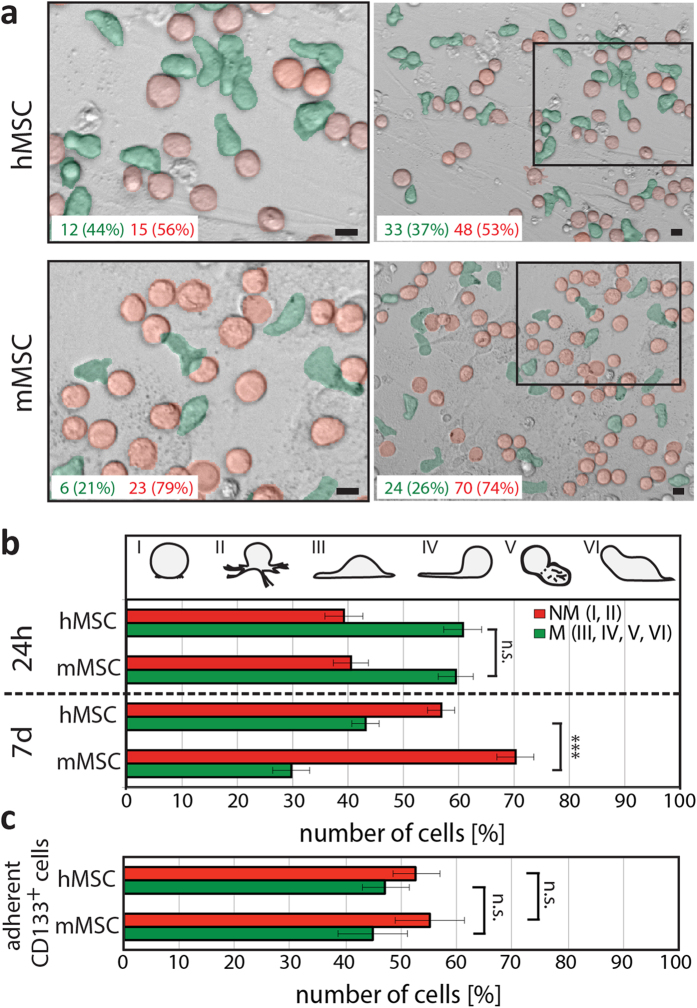
The relative amount of human HSPCs exhibiting a migrating morphology is reduced on murine MSCs in comparison to human ones. (**a**) Phase contrast pictures of human (h) HSPCs growing on either h (top panels) or murine (m; bottom panels) MSCs for 7 days. The absolute amounts of migrating and non-migrating hematopoietic cells, which have an elongated (green) or spherical (red) morphology, respectively, observed in each frames are indicated. Left panels show higher magnification views of areas indicated in the corresponding images on the right. (**b,c**) Numbers of hematopoietic cells exhibiting morphologies characteristic of non-migrating (NM, red) and migrating (M, green) cells, respectively (see Fig. 2), were counted after 24 hours and 7 days of co-culture on MSCs (**b**). Numbers CD133^+^ hHSPCs with a given morphology after 7 days are indicated (**c**). Three to four experiments were performed using CD34^+^ hHSPCs isolated from different donors, and at least 400 (**b**) or 100–200 (**c**) cells were analyzed per condition. Three asterisks indicate a significant difference as calculated by two-tailed paired student’s t-test (*p* < 0.001). N.s., not significant (*p* ≥ 0.05). Scale bars: 10 μm.

Their migratory behavior was recorded using time-lapse video and cellular tracking (Fig. 2b). After 24 hours or 7 days of co-culture, the average migration distances of hematopoietic cells are similar independent of the species origin of the MSCs (Fig. 2c,d). However, we noticed during video recording that numerous hematopoietic cells were not moving, but maintain a spherical morphology typical of non-migrating cells (Fig. 3a). This prompted us to quantify the ratio of migrating and non-migrating cells. Surprisingly, the proportion of cells harboring a migratory morphology is significantly reduced when they were cultured for 7 days on mMSCs in comparison to their human counterparts (Fig. 3b). Such difference is not observed within the first 24 hours of cultivation (Fig. 3b). The presence (or absence) of CD133 did not change the ratio of migrating versus non-migrating cells (Fig. 3c), as previously reported^30^, indicating that other factors in these cell-based systems influence the hematopoietic cell behavior. Thus, our data suggest that hHSPCs are migrating similarly on both m and hMSCs, but the proportion of migrating cells is significantly reduced on murine feeder cells.

### Adhesion strength of human hematopoietic stem and progenitor cells to the mesenchymal stromal layers

**Figure 4:**
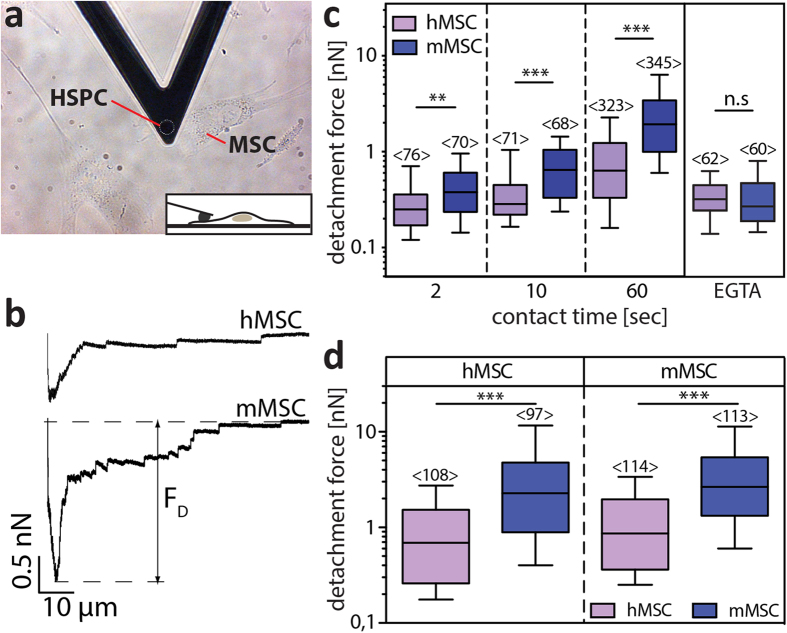
The adhesion forces of human HSPCs on murine or human MSCs are measured by AFM. (**a**) Phase contrast image of a human (h) HSPC attached to a tipless AFM cantilever. MSCs growing on fibronectin-coated coverslips are visible (see Supplementary Fig. S1). The inset illustrates a side-view of the contact between a single hHSPC and a MSC. (**b**) Representative retract F-D curves of an adhesion measurement between hHSPC and h (top panel) or murine (m, bottom panel) MSC are depicted. The contact time was 60 sec. The minimum of the curves (with respect to the zero force level) is the maximum detachment force (F_D_) needed to separate both cells. (**c**) Box-whisker plots show F_D_ of freshly isolated CD34^+^ hHSPC measured from h (purple bars) or m (blue bars) MSC after a contact time of 2, 10 or 60 sec in absence or presence of 5 mM EGTA (EGTA, 60 sec contact time). (**d**) Box-whisker plots show F_D_ of CD34^+^ HSPCs pre-cultured for seven days either on h (left panel) or m (right panel) MSCs prior to force measurements on h (purple bars) or m (blue bars) MSC with a contact time of 60 sec. Box-whisker plots represent half of the data points within the box and 80% within the whiskers. Horizontal lines within the box represent median values. Numbers within bars (<*n*>) show the total number of analyzed F_D_ curves. Four experiments were performed for each condition using CD34^+^ hHSPCs isolated from different donors. MSCs were derived from one donor or one murine cell preparation, but additional data with other donors/cell preparations are presented in Supplementary Fig. S6. The pairwise comparison of data was performed using Mann-Whitney-U-test (**p < 0.01; ***p < 0.001; n.s., not significant, ≥0.05).

The phenotypic difference observed upon expansion of hHSPCs on m *versus* h feeder cell layers (Fig. 1a) and the divergence in the ratio of migrating and non-migrating cells (Fig. 3) prompted us to compare their adhesion strength to MSCs by AFM-based SCFS. The experiments were performed using CD34^+^ hHSPCs freshly isolated or pre-cultured on MSCs for 7 days (see Methods). Technically, a single adherent hematopoietic cell was attached to a WGA-coated AFM-cantilever and approached towards a MSC (Fig. 4a). After a defined contact time (2, 10 or 60 sec) the cantilever-bound hematopoietic cell was retracted. Adhesive interactions that have formed during the contact bent the cantilever downwards. Once the restoring force of the cantilever exceeded the strength of the interactions between these cells, the hHSPC started to detach from the substratum-bound MSC. During the approach and retraction phase the bending of the cantilever, which corresponds to the force acting on it, is recorded and can be plotted as a force-distance (F-D) curve (Fig. 4b). The minimum of the retraction curve (with respect to the zero force level) corresponds to the maximum detachment force needed to detach both cells (F_D_; Fig. 4b). Surprisingly, the median F_D_ of CD34^+^ hHSPC from mMSC after a contact time of 60 sec was about threefold higher than from their human counterpart (Fig. 4c). Similar F_D_ were observed using hHSPCs that were pre-cultured for 7 days either on m or hMSCs, suggesting that differential adhesion properties between m and hMSCs were conserved upon culture and expansion of hHSPCs (Fig. 4d). Comparable results are obtained using hMSCs derived from different donors or mMSCs from different isolation preparations (Supplementary Fig. S6). The passage number (3–5/4–20 for h/mMSCs, respectively) and the confluence had no influence on HSPC-MSC interaction (data not shown). Nonetheless, we found a drastic reduction of ≈80/60% of the F_D_ between hHSPCs and m/hMSCs, respectively, when a chelating agent (EGTA, 5 mM) was added to the medium during the recording of the adhesive interactions (Fig. 4c, EGTA). This indicates that the major interactions between hHSPCs and MSCs are dependent on calcium ions. The presence of EGTA did not affect the adhesion of the MSCs to the substratum or their spindle shape under the condition used for AFM-based SCFS (Supplementary Fig. S7).

Next, we attempted to determine the impact of glycosylation of MSCs on the adhesion of hHSPCs given that the binding of numerous lectins to certain sugars is dependent on calcium (Ca^2+^). Unfortunately, the pre-incubation (1 μg/μl, 20 hours, 37 °C) of m and hMSCs with tunicamycin, an inhibitor of N-glycosylation, strongly reduced their adhesion to the substratum (data not shown), which impeded the AFM analysis.

### N-cadherin mediated interaction of human hematopoietic stem and progenitor cells with murine and human multipotent mesenchymal stromal cells

**Figure 5:**
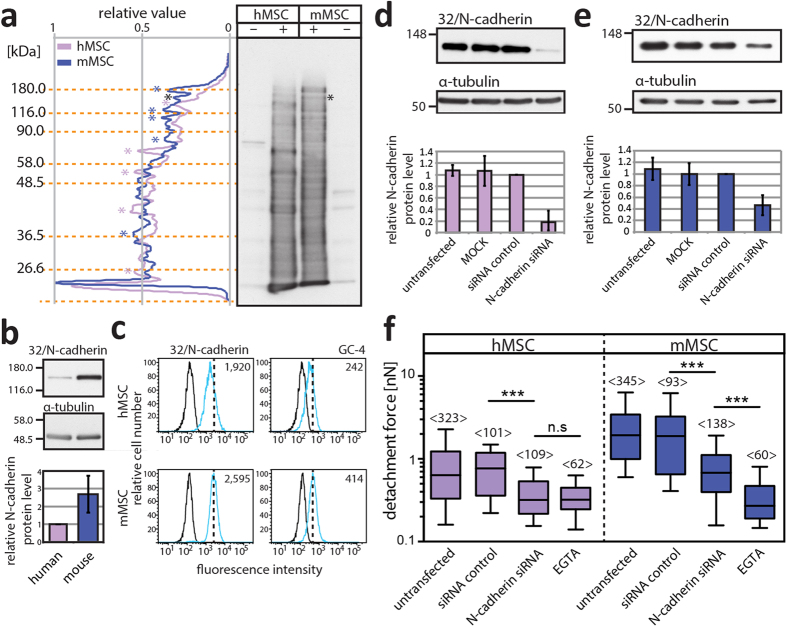
Mesenchymal cell-associated N-cadherin is the major player in the differential adhesion of human HSPCs. (**a**) Human (h) or murine (m) MSCs were incubated without (–) or with (+) sulfo-NHS-LC-biotin prior to solubilization. Biotinylated proteins were detected using HRP-conjugated streptavidin. The purple and blue asterisks indicate the major cell surface proteins in h and mMSCs, respectively. The black ones indicate the potential N-cadherin signal. (**b**) Expression level of N-cadherin in h *versus* mMSCs. N-cadherin was detected by immunoblotting using 32/N-cadherin antibody and its expression was normalized to α-tubulin. Values are relative to that of hMSC (*n* = 5, MSCs were derived from three distinct donors or murine preparations). (**c**) Flow cytometry analysis of PFA-fixed, saponin-permeabilized cells labeled with two distinct N-cadherin antibodies (clones 32/N-cadherin and GC-4) followed by an anti-mouse DyLight^®^649-conjugated secondary antibody. The antigen expression (cyan) and the appropriate isotype control (black) are shown. The mean fluorescence intensity appears in the right-top corner. (**d**,**e**) Human (**d**) or m (**e**) MSCs were either untransfected or transfected without (MOCK) or with negative control or N-cadherin siRNAs. N-cadherin knockdown was confirmed 20 hours after transfection by immunoblotting, and its expression normalized to α-tubulin (*n* = 3). (**f**) Box-whisker plots show F_D_ of freshly isolated CD34^+^ HSPC from h (purple) or m (blue) MSC measured 16–20 hours after transfection with a contact time of 60 sec. For comparison, the data obtained with untransfected MSCs and EGTA were presented (Fig. 4). For each transfection experiments (*n* = 3), CD34^+^ HSPCs were derived from distinct donors. Box-whisker plots represent half of the data points within the box and 80% within the whiskers. Horizontal lines within the box represent median values. Numbers within bars (<*n*>) show the total number of analyzed F_D_ curves. The pairwise comparison of data was performed using Mann-Whitney-U-test (***p < 0.001; n.s., not significant, *p* ≥ 0.05).

The collected results prompted us to evaluate the general profile of cell surface glycoproteins expressed on mMSCs in comparison to their human counterparts. This investigation might give a hint about a particular set of proteins involved in the differential adhesion of HSPCs. Surface biotinylation was performed and biotinylated proteins were detected with streptavidin. Although their expression profiles are highly complex a certain similarity appears between them (Fig. 5a). Some proteins were strongly expressed in murine cells and others in human ones (Fig. 5a, blue and purple asterisk, respectively). It will be of interest to identify them by a proteomic approach. One biotinylated protein, strongly expressed in mMSCs, caught our attention since its molecular mass (130 kDa) corresponds to N-cadherin (Fig. 5a, black asterisk). This molecule is known to be an important player in the interaction of HSPCs with the endosteal niche and belongs to the cadherin superfamily of Ca^2+^-depended cell-cell adhesion molecules^19^,^20^. We analyzed its expression by immunoblotting. Mouse MSCs showed a 2.6-fold higher amount of N-cadherin as their human counterparts (Fig. 5b). The mean fluorescence intensity observed by flow cytometry using two distinct anti-N-cadherin antibodies is also higher in murine cells (Fig. 5c). The elevated N-cadherin expression might explain the differential adhesion strength of hHSPCs, which was further investigated using a siRNA-mediated knockdown approach (Fig. 5d,e). Interestingly, the reduction of N-cadherin by 82.1 ± 19.4% in hMSCs (Fig. 5d) led to a ≈42% drop of the F_D_ in comparison to cells transfected with negative siRNA control (Fig. 5f, left panel). Surprisingly, F_D_ of 0.3 nN corresponds to the one obtained when EGTA was added (see also Fig. 4c) suggesting that most, if not all, Ca^2+^-dependent intercellular interaction rely on this molecule. In mMSCs, the siRNA-mediated 53.6 ± 17.3% reduction of N-cadherin led to a decrease ≈36% of the F_D_ of hHSPCs (Fig. 5f, right panel). The remaining N-cadherin might explain the difference in F_D_ observed in presence of EGTA (Fig. 5f, right panel). By coincidence, the amount of N-cadherin in N-cadherin-siRNA transfected mMSCs reached a comparable level to untransfected hMSCs, and the resulting F_D_ of hematopoietic cells are in a similar range (0.68 and 0.77 nN, respectively) suggesting that the N-cadherin expression level in MSCs explains, at least in part, the differential adhesion strength of hHSPCs on m *versus* hMSCs.

## Discussion

We investigated the hematopoiesis-supporting properties of BM-derived MSCs obtained from different species. We found that mMSCs support the proliferation and migration of CD34^+^ hHSPCs in agreement with recent reports demonstrating the expansion of CD34^+^ hematopoietic cells in the presence of either murine stromal cell lines or mMSC-like cells derived from dental pulps^31^,^32^. Nonetheless, we observed subtle differences in their capacity to support certain hHSPC subpopulations and to maintain the HSPC polarization by comparing them to those derived from human tissues.

First, the *ex vivo* expansion of hHSPCs with primitive phenotypes (CD34^+^CD133^+^ and CD133^+^CD34^–^) is significantly lower on murine feeder cells, which might explain the reduced number of nucleated cells after one week of co-culture. Indeed, the CD133^+^ hematopoietic cells expanded on murine cells in the same way as those growing on fibronectin, suggesting that factors secreted by hMSCs and/or specific interaction(s) between human cells are altered in the heterologous system. This observation is consistent with the enhanced engraftment of cord blood-derived CD34^+^ hHSPCs and accelerated hematopoietic recovery when they are co-transplanted with hMSCs (or other supporting cells such as osteoblasts) in non-obese diabetic/severe combined immunodeficiency mice^33^,^34^. A quantitative differential gene expression profile of m *versus* hMSCs might be useful to identify essential players involved in the proliferation of CD133^+^ HSPCs particularly those with a CD133^+^CD34^–^ phenotype^4^,^7^,^15^,^28^. The latter cells might have specific lineage potential^26^,^27^,^28^, and hence a particular contribution to the lineage specification of human hematopoietic tree^35^. It might be more than a coincidence that CD133 has the possibility to be symmetrically or asymmetrically distributed in dividing CD34^+^ HSPCs and other stem cells, and extrinsic factor(s) influencing these division modes could arise from the proper and species-specific microenvironment^36^,^37^. Likewise, the early release of CD133^+^ membrane vesicles by hematopoietic cells cultured on mMSCs, which is a sign of cell differentiation^6^, may indicate that stemness features are more rapidly lost when an interspecies cellular interaction is engaged. The phenomenon seems to result from specific interactions given that the release of CD133^+^ membrane vesicles is not stimulated when hHSPCs are cultured on fibronectin^6^. The interaction of hHSPCs with the murine cells might promote their rapid differentiation, but further investigations are needed to determine the exact impact of feeder layers on hematopoietic differentiation^7^. Exploring these issues is important since transplantation of hHSPCs into murine models failed to produce all functional blood cells^38^. The implication of CD133 itself in murine hematopoiesis might be discrete as demonstrated in healthy CD133-null mice, but might play a role in the hematopoietic system after myelotoxic stress^30^.

Second, hHSPCs growing on mMSCs, just like on human cells^4^, develop numerous types of membrane protrusions and migrate in a similar way indicating that essential interactions with feeder cells are conserved across species^16^. Surprisingly, the number of migrating cells is reduced upon co-culture on mMSCs pointing again to certain factors, soluble/membrane-bound, being limited (or absent) in the murine system. Such limitation might have certain consequence in the homing of circulating HSPCs particularly post-transplantation (see below). We may assume that intercellular communication between the HSPCs and MSCs is perturbed when they are derived from different species^39^,^40^. It is documented that hematopoietic progenitors regulate their microenvironments through the direct transfer of proteins and lipids via a proper membrane contact between the uropod of migrating hHSPCs and supporting stromal cells and/or via the transfer of hematopoietic cell-derived membrane vesicles^6^,^41^, which are released specifically during their differentiation process (see above)^6^. Upon internalization, transferred materials can modify the gene expression profile of supporting stromal cells resulting in the production of cell-signaling molecules, notably stromal-derived factor-1, a factor involved in hematopoietic migration^41^. Excitingly, CD133 has been used to highlight both mechanisms of HSPC–stromal cell communication^6^,^41^, and we may hypothesize a feedback mechanism where CD133^+^ hHSPCs might specifically exchange information with human stromal cells that in turn would regulate their proliferation, self-renewal and migration.

To decipher the subtle difference observed in hematopoiesis-supporting properties, we compared the adhesion strength of hHSPCs on MSCs by AFM-based SCFS. This method is extremely versatile and allows quantifying cell adhesion forces in spatially and temporally controlled conditions^42^,^43^. We observed a quantitative difference in the adhesion strength of hHSPCs on stromal cells suggesting that the affinity of certain receptor-ligand and/or adhesive interactions or the expression levels in a given cellular partner differ when an inter-species system is engaged. Whether the enhanced adhesion of hHSPCs on murine cells is the sole physical factor, and directly involved in subtle phenotypic and morphological differences observed upon the expansion of hHSPCs needs further investigation. However such singularity may help to explain why in xenogenic murine recipients human stem cells are predominantly found within the BM and secondary lymphoid organs after transplantation, rather than in the peripheral blood^44^. Although the expression profile of cell surface molecules between BM-derived m and hMSCs seems to be similar (Fig. 5a, Supplementary Figs S2 and S3)^45^, their complete cell surface proteome might highlight all candidate proteins that are responsible for the divergence in the HSPC adhesion property^21^. Focusing on the Ca^2+^-depended adhesion protein N-cadherin, we found that its differential expression between m and hMSCs can explain, at least in part, the strong adhesion of hHSPCs on murine cells (Fig. 5). Previous studies using species-specific homophilic interactions have suggested the role of stromal cell-associated N-cadherin in *ex vivo* proliferation of hHSPCs^5^,^20^. In mouse, spindle-shaped N-cadherin^+^ osteoblasts located in the endosteum appear to be an important player of the HSPC niche^46^,^47^,^48^. Therein, osteoblast-associated N-cadherin tethers HSPCs to the niche and keeps them in a quiescent state through modulation of β-catenin signaling. Incidentally, diverse cellular pathways link the expression of CD133 to β-catenin signaling and a correlation between the membranous localization of β-catenin and the release of CD133^+^ membrane vesicles might exist^49^. The implication of N-cadherin in cells of osteolineage was challenged using conditional N-cadherin gene ablation or osteoblast depletion in biglycan-deficient mice^50^,^51^,^52^. It cannot be excluded that other cell types, notably MSCs (e.g., nestin^+^, this study and Ref. 14), or alternative cadherin family members are compensating for the loss of osteoblast-associated N-cadherin in BM niches of these murine models. Irrespective of the current debate, the high expression of N-cadherin in mMSCs might interfere with the physiological expansion and maintenance of human cells in mice suggesting that not only the presence or absence of a given interacting player is significant but also its expression level, which raises a novel dimension in the understanding of the BM niche. Our data obtained with HSPCs could be extended to cancerous ones (i.e. cancer stem cells) when murine transplantation models are used^53^. It might be worth to evaluate the homing and engraftment of hHSPCs as well as the behavior of human cancer cells in a murine model with lower N-cadherin expression (e.g., N-cadherin^+/–^ mice).

When AFM-based SCFS experiments were performed with non-adherent hHSPCs we noted that their binding to WGA-coated AFM-cantilever is highly reduced (data not shown), suggesting that the glycosylation profile of non-adherent cells is distinct to that of adherent ones. Unfortunately, the lack of binding to the cantilever impedes the adhesive force measurement. It will be of interest to probe distinct subpopulation of hHSPCs according to their expression of stemness markers (CD34, CD133) with various lectins. Indeed, Hemmoranta and colleagues have shown that N-glycan structures of cord blood-derived CD133^+^ and CD133^–^ cells are distinct^54^. Further studies are urged to determine quantitatively the impact of hematopoietic-associated N-cadherin or other glycoproteins (e.g., integrin beta-like 1) and/or glycolipids (e.g., gangliosides) on HSPC–MSC interaction^7^,^55^. Indeed, the differential lectin binding sites observed between m and hMSCs (Supplementary Fig. S4, Table S1) and the selective expression of certain gangliosides (i.e., GM1 and GM3, Supplementary Fig. S5) will deserve particular attention in a near future. The AFM-based SCFS investigations could be extended to other BM components^56^, and the determination of the adhesion properties of cancerous cells that originate from early hematopoietic stem cells or metastatic cancer cells might be of clinical interest.

## Conclusion

MSCs derived from murine BM are capable of supporting hHSPCs proliferation and migration *ex vivo*. Nonetheless, subtle differences in expanded hHSPCs were observed by comparison to their growth on human cells. The differential HSPC adhesion properties of m *versus* hMSCs caused by N-cadherin among other molecular players might explain these differences. This observation should be kept in mind when murine transplant models are employed to analyze human stem cell biology.

## Methods

### BM-derived mesenchymal stromal cells

Primary mMSCs were obtained from 6–10 week-old C57BL/6 mice as described^18^, and isolated cells were cultured in MSC medium [DMEM-low glucose supplemented with 10% fetal calf serum (FCS)] as detailed described in the Supplementary Methods. They were used between passage 4 and 21. Animal experiments were performed in strict accordance with German Animal Welfare legislation. Mice were kept and bred at the Animal Facility of Max-Planck-Institute of Molecular Cell Biology and Genetics (Dresden), which holds necessary licenses (74–9165.40–9–2000-1 and 74–9168.25–9–2001–1) for keeping laboratory animals and collecting organs and tissues, both issued by Regierungspräsidium Dresden, Saxony.

Primary hMSCs were extracted from BM aspirates collected from healthy donors after verbal and written consent. The study was approved by the local ethics committee (Ethikkommission an der Technischen Universität Dresden, Ethic board no. EK263122004). Plastic-adherent hMSCs were obtained and cultured in MSC medium as described^4^,^23^. They were used between passage 3 and 5.

Both m/hMSC cultures were passaged upon reaching 70–80% confluence, and reseeded at a density of 2–5 × 10^3^ cells per cm^2^ of surface area. Under these conditions, MSCs took around 7 (±2) days to reach confluence and maintained their proliferative and differentiation capacities.

### Differentiation of mesenchymal stromal cells

The adipocyte and osteogenic differentiation of MSCs were performed as described previously^23^, and they were evaluated by Oil red O and Alizarin Red S (Sigma-Aldrich) staining, respectively, as documented in the Supplementary Methods.

### Colony-forming unit–fibroblast assay

For the colony-forming unit–fibroblast (CFU-F) assay, 250 MSCs were seeded on 35-mm dishes and cultured in MSC-medium for 2 weeks. After fixation with 100% methanol, cells were stained with a Giemsa’s azur eosin methylene blue solution (Merck) for 30 min. Adherent cell clusters containing >50 cells were counted as a colony.

### Hematopoietic stem and progenitor cells and co-cultivation with mesenchymal stromal cells

hHSPCs were collected from healthy donors after informed consent and approval of the local ethics committee (Ethic board no. EK201092004) and isolated from mobilized peripheral blood directly after leukapheresis by immunomagnetic separation based on CD34 as described^4^. Mobilization was achieved by subcutaneous injection of granulocyte colony-stimulating factor (G-CSF; 7.5 μg/kg per day; Granocyte, Chugai Pharma)^4^.

CD34^+^ hHSPCs were cultured on either confluent m or hMSCs in HSPC medium [serum-free medium (CellGro SCGM, CellGenix) supplemented with early-acting cytokines (50 ng/ml stem cell factor, 50 ng/ml Fms-related tyrosine kinase 3 ligand (CellGenix), 15 ng/ml interleukin-3 (R&D Systems))] at a density of 7.5 × 10^4^ and 6 × 10^3^ cells per cm^2^ for one and seven days, respectively, in a humidified 5% CO_2_ atmosphere at 37 °C. [Under serum-free conditions, the proliferation of MSCs is strongly reduced, and the addition of cytokines neither alters it nor their general morphology. Moreover, we did not use MSC cultures with more then 2 days post-confluence since the cell monolayers were occasionally detaching after 7 days in co-cultivation with hHSPCs]. Alternatively, the freshly isolated CD34^+^ hHSPCs were kept in HSPC medium without MSCs at 37 °C in order to recover from magnetic cell separation. Cells were incubated 16–24 hours prior AFM analysis. Data obtained within this time window are comparable (data not shown).

### Short interfering RNA transfection

MSCs (7 × 10^5^) were transfected by electroporation with 1,5 μg of N-cadherin siRNA or negative control (low GC content) siRNA (Invitrogen, oligonucleotide IDs HSS101671 and 12935–200, respectively) or without siRNA (MOCK) according to the manufacturer’s instructions using optimized protocol for hMSCs (VPE-1001, Lonza). Transfected cells were seeded in pre-warmed medium at a density of 1 × 10^4^ cell per cm^2^, and cultured 14–24 hours prior to use.

### Cell-surface biotinylation

All steps were conducted at 4 °C. The membrane-impermeable sulphosuccinimidyl-6(biotinamido)-hexanoate biotinylating agent (called EZ-Link® Sulfo-NHS-LC-Biotin, Thermo Scientific) was dissolved in Ca/Mg-PBS (phosphate-buffered saline containing 1 mM CaCl_2_, 0.5 mM MgCl_2_) to a final concentration of 0.2 mM. After repeated washes with Ca/Mg-PBS, subconfluent MSCs (on 35-mm dishes) were incubated with 1 ml of biotin solution for 30 min, washed three times with Ca/Mg-PBS, incubated with Ca/Mg-PBS containing 20 mM glycine for 10 min and lysed in solubilization buffer. Detergent extracts were analyzed by SDS-polyacrylamide-gel electrophoresis (PAGE), and biotinylated cell surface proteins were detected with horseradish peroxidase (HRP)-conjugated streptavidin (Thermo Scientific). Their profile was obtained using Image J^57^.

### Protein extraction and immunoblotting

Proteins were separated by SDS-PAGE and analyzed by immunoblotting as described in Supplementary Methods. Membranes were probed with N-cadherin (clone 32/N-cadherin, BD Bioscience), CD133 (80B258^58^), β-actin (AC-40, Sigma-Aldrich) or α-tubulin (DM1A, Sigma-Aldrich) mouse IgG1 antibodies. The anti-N-cadherin antibody recognized both mouse and human proteins while the antibody derived from clone 80B258 does not cross-react with murine CD133^58^.

### Flow cytometry

MSCs were harvested by a treatment with 0.05% trypsin/0.5 mM EDTA solution (Life Technologies) for 3–5 min, or alternatively with a Collagenase IV solution (100 U/μl; diluted in Hanks balanced salt solution (Life Technologies) containing 1 mM CaCl_2_, 0.5 mM MgCl_2_) for 30 min at 37 °C. [The presence of calcium in the dissociation medium protects Ca^2+^-dependent cell adhesion molecules against proteolytic cleavage]. Cells were centrifuged and the pellet resuspended in PBS containing 2% FCS (PAA Laboratories). Cell suspensions (5 × 10^5^ cells in 100 μl) were incubated for 30  min at 4 °C with either fluorochrome-conjugated or unconjugated primary antibodies (Supplementary Table S2). For the detection of nestin, vimentin, and N-cadherin, paraformaldehyde (PFA)-fixed cells were permeabilized with 0.2% saponin (AppliChem) prior to labeling. Unconjugated antibodies were detected with the appropriate secondary antibody: donkey anti-rat DyLight^®^649, anti-rabbit DyLight^®^649, anti-goat Alexa Fluor^®^633 or anti-mouse DyLight^®^649 (Jackson ImmunoResearch Inc). GM1 was detected by Alexa Fluor^®^488-conjugated Cholera Toxin Subunit B (Life Technologies). After washing with PBS, 20,000 events were acquired on a LSRII (BD Bioscience). Instrument settings and gating strategies were established using isotype controls. Data were analyzed using Diva (BD Bioscience) and FlowJo (TreeStar) software. hHSPCs were labeled with fluorochrome-conjugated primary antibodies (Supplementary Table S3) and gated on CD45^+^ population.

### Lectin labeling

The cell-surface labeling of MSCs with 20 different lectins were performed as described^23^. Cells were grown on fibronectin-coated glass coverslips and labeled for 30 min at 4 °C with fluorescein isothiocyanate-conjugated lectins (Vector Laboratories) (see Supplementary Table S1). After washing with PBS, labeled cells were fixed with 4% PFA for 20 min at room temperature, and quenched with 50 mM NH_4_Cl containing 4,6-diamidino-2-phenylindole (DAPI, Life Technologies) for 10 min. Coverslips were rinsed with PBS and distilled water, and mounted in Moviol 4.88. Cells were observed using a Leica SP5 upright confocal laser scanning microscope.

### Immunofluorescence and confocal microscopy

Confluent MSC-monolayer growing on fibronectin-coated glass coverslips were labeled with primary antibodies specified in Supplementary Table S2. After sample processing as documented in the Supplementary Methods, images were captured using a Leica SP5 upright confocal laser scanning microscope. All images shown were processed with Adobe Photoshop software.

### Time-lapse microscopy and scanning electron microscopy

Time-lapse recording of migrating hHSPCs was performed as described^17^. Samples for the SEM analyses were prepared as previously described^4^. Technical details are provided in the Supplementary Methods.

### Differential centrifugation of membrane vesicles

Conditioned medium (3 ml) recovered after 7 days of HSPC–MSC co-culture was subjected to differential centrifugation as follows: 5 min at 300 × *g*; 20 min at 1,200 × *g*; 30 min at 10,000 × *g* and 1 hour at 200,000 × *g*. Adherent hematopoietic cells were harvested by flushing them off the feeder layer with ice-cold PBS, and centrifuged 5 min at 300 × *g.* The 300 × *g* pellets were lysed with solubilization buffer as described above while pellets of 1,200, 10,000 and 200,000 × *g* centrifugation were resuspended in Laemmli buffer and analyzed by immunoblotting for CD133 and actin.

### Atomic force microscopy-based single-cell force spectroscopy

A NanoWizard II AFM equipped with the CellHesion module (JPK Instruments), mounted on an inverted light microscope (Axiovert 200, Carl Zeiss) was used to perform AFM-based SCFS. All measurements were performed at 37 °C using a temperature-controlled sample chamber (PetriDish Heater, JPK Instruments) and more details are provided in Supplementary Methods. Tipless cantilevers having a nominal spring constant of 0.08 N/m (PNP-TR-TL-Au, Nanoworld) were coated with 1 mg/ml WGA (Vector Laboratories) as described^59^. Cantilevers were calibrated before every experiment using build-in procedures of the AFM software (JPK Instruments) based on the equipartition theorem^60^. Before SCFS experiments, MSCs were grown on either fibronectin-coated 30-mm Petri dishes (TPP) (protocols a and b, see Supplementary Fig. S8) or fibronectin-coated coverslips until reaching confluence (protocol c, Supplementary Fig. S8). The latter protocol is easier to handle when numerous samples are analyzed. For SCFS experiments, coverslips were placed in 30-mm Petri dishes containing measurement medium (HSPC medium containing 50 mM HEPES). For investigating adhesion forces in the absence of calcium, 5 mM Ca^2+^-chelating agent EGTA (BDH Chemicals) was added to the media. Subsequently, 1 × 10^3^ CD34^+^ hHSPCs, freshly isolated (see above) or pre-cultured for seven days on MSCs, were seeded into the Petri dish. A single HSPC was attached to the AFM cantilever as described^59^. Pre-cultured adherent hematopoietic cells were obtained by flushing the HSPC–MSC co-culture after two gentle washing steps in order to remove non-adherent ones first. Under these conditions, ≈90% of adherent cells were positive for CD34 (*n* = 3, data not shown).

For force-distance (F-D) curve measurements, the cantilever-bound HSPC was approached at a constant speed of 5 μm/sec onto a MSC until a contact force of 1 nN was reached. Before retraction of the cantilever-bound cell at constant speed (5 μm/sec), cells were maintained in contact for 2, 10 or 60 sec in constant height mode. Typically 60-345 F-D curves generated from at least 10 cells from 3 individual donors were analyzed per MSC preparation and contact time. The data processing software provided by the AFM manufacturer (JPK Instruments) was used to extract the maximum detachment force (F_D_) from F-D curves.

### Statistical analysis

Data are expressed as the mean ± standard deviation of at least three independent experiments. Box and-whisker plots represent the values between the lower and upper quartile within the box and either maximum and minimum (Fig. 2) or 80% (other figures) within the whiskers. Horizontal lines within the box represent median values. Statistical analyses were performed using either two-tailed paired student’s t-test or the non-parametric Mann-Whitney-U-test when data (i.e. cell detachment forces recorded by SCFS) were not Gaussian distributed. Observed differences were regarded as significant if the calculated two-tailed *p*-values were ≤ 0.05. *p < 0.05, **p < 0.01, ***p < 0.001. All analyses were performed using GraphPad Prism 6 (GraphPad Software Inc., USA).

## Additional Information

**How to cite this article**: Reichert, D. *et al.* Phenotypic, Morphological and Adhesive Differences of Human Hematopoietic Progenitor Cells Cultured on Murine versus Human Mesenchymal Stromal Cells. *Sci. Rep.* **5**, 15680; doi: 10.1038/srep15680 (2015).

## Supplementary Material

Supplementary Information
