# Supplementary material for: Phenotypic, Morphological and Adhesive Differences of Human Hematopoietic Progenitor Cells Cultured on Murine versus Human Mesenchymal Stromal Cells
Source: Sci Rep. 2015 Oct 26;5:15680. doi: 10.1038/srep15680 (PMC4620509; doi:10.1038/srep15680)
Supplement: Supplementary Information [file srep15680-s1.pdf]

Phenotypic, Morphological and Adhesive Differences of Human Hematopoietic Progenitor Cells Cultured on Murine versus Human Mesenchymal Stromal Cells

Doreen Reichert[1,¶], Jens Friedrichs[2,¶], Steffi Ritter[1], Theresa Käubler[1], Carsten Werner[2,4], Martin Bornhäuser[3,4], and Denis Corbeil[1,4,*]

[1] Tissue Engineering Laboratories (BIOTEC), Technische Universität Dresden, Dresden, Germany

[2] Institute for Biofunctional Polymer Materials, Leibniz Institute of Polymer Research Dresden, Dresden, Germany

[3] Medical Clinic and Polyclinic I, University Hospital Carl Gustav Carus, Dresden, Germany

[4]DFG Research Center and Cluster of Excellence for Regenerative Therapies Dresden, Dresden, Germany

¶Both authors contributed equally

*Correspondence: Denis Corbeil

Phone: 49-351-463-40118

Fax: 49-351-463-40244

E-mail: denis.corbeil@biotec.tu-dresden.de

List of contents:

Supplementary Methods
Supplementary References
Supplementary Figures (S1 to S8)
Supplementary Tables (S1 to S3)

Supplementary Methods

Isolation and culture of murine bone marrow-derived mesenchymal stromal cells

The 6-10 week-old C57BL/6 mice were kept and bred at the Animal Facility of Max-Planck-Institute of Molecular Cell Biology and Genetics (Dresden), which holds necessary licenses (74-9165.40-9-2000-1 and 74-9168.25-9-2001-1) for keeping laboratory animals and collecting organs and tissues, both issued by Regierungspräsidium Dresden, Saxony. Animal experiments were performed in strict accordance with German Animal Welfare legislation.

Three animals were sacrificed for each mesenchymal stromal cell (MSC) preparation, and three independent cell isolations were performed as described[1]. Each preparation generated similar data with the exception of CD90.2 expression (see below or Results). Briefly, CO_2-mediated anesthetized mice were killed by cervical dislocation, and femurs and tibias were dissected. All surrounding tissues were carefully removed, and the ends of the bones were cropped. Subsequently, bones were crushed with a pestle in ice-cold Hanks-balanced salt solution (HBSS, Life Technologies) supplemented with 2% fetal calf serum (FCS, PAA laboratories), 1% penicillin/streptomycin (Life Technologies), 2% D-glucose (VWR). The crushed bones were gently washed once in HBSS, and the cell suspension filtered through a 70-µm cell strainer was discarded. The bone fragments were collected and incubated for 1 hour at 37ºC in 20 ml of Dulbecco's modified Eagle medium (DMEM)-low glucose containing 0.2% collagenase (type: CLS1, Biochrom GmbH), 10 mM Hepes and 1% penicillin/streptomycin. The suspension was filtered with a 70-µm cell strainer to remove bone fragments and debris, and cells were collected by centrifugation at 300 x *g* for 5 min at 4ºC. Isolated cells were cultured in MSC medium [DMEM-low glucose supplemented with 10% FCS] in a humidified 5% CO_2 atmosphere at 37ºC. We added a low concentration of human basic fibroblast growth factor (3 ng/ml, Miltenyi Biotec) to

facilitate the initial cell expansion[2]. The differential level of N-cadherin expression between the m and hMSCs is not modified under this condition (data not shown).

Differentiation of mesenchymal stromal cells

To induce adipocyte differentiation, subconfluent MSCs were cultured on 6-well plates for 2 weeks in adipogenic medium [MSC-medium supplemented with 500 µM isobutyl-methyl xanthine, 1 µM dexamethasone, 10 µM insulin, and 200 µM indomethacin (all purchased from Sigma-Aldrich)] with medium exchange twice a week. The adipogenic differentiation was evaluated by labeling the intracellular lipid droplets with Oil red O (Sigma-Aldrich). In brief, cells were fixed with 4% paraformaldehyde (PFA) for 30 min at room temperature, rinsed twice with deionized water, and dehydrated with 100% 1,2-propanediol (Carl Roth) for 3 min at room temperature. The plates were gently mixed. Afterward, cells were stained with preheated Oil Red O solution (0.5% in 1,2-propanediol) for 8 min in a 60ºC oven. The labelling solution was partly removed, and 85% 1,2-propanediol was added for 3 min at room temperature. Cells were rinsed twice with deionized water and observed under an inverted light microscope.

For osteogenic differentiation, subconfluent MSCs were cultured for 3 weeks in osteogenic medium [MSC-medium supplemented with 10 mM β-glycerophosphate, 0,1 µM dexamethasone, and 200 µM ascorbic acid (all purchased from Sigma-Aldrich)]. To identify calcium deposits in culture, 4% PFA-fixed cells were rinsed twice with distilled water and stained with filtered 2% Alizarin Red S (Sigma-Aldrich) dissolved in distilled water (pH 4.2) for 30 min at room temperature in the dark. The remaining dye was washed out with distilled water, and the cells were washed once more prior imaging.

Protein extraction and immunoblotting

MSCs were scraped, centrifuged and lysed in solubilization buffer (1% NP-40, 0.5% deoxycholate, 0.1% sodium dodecylsulfate, 150 mM NaCl, 50 mM Tris-HCl, pH 8.0) supplemented with complete protease inhibitor (Roche) at 4°C. Detergent extracts obtained after centrifugation (10 min, 10,000 x *g*) were used for immune and biotin-streptavidin blotting. Proteins were separated by SDS-PAGE (7.5%) and transferred to a poly(vinylidene fluoride) membrane (Millipore, pore size 0.45 µm)[3]. Membranes were incubated in blocking buffer (PBS containing 0.3% Tween 20 and 5% milk powder (general protocol) or 3% bovin serum albumin (for the detection of biotinylated proteins)) overnight at 4°C prior to being probed with a primary antibody for 1 hour at room temperature. Antigen-antibody complexes were detected using horseradish peroxidase-conjugated secondary antibody (Jackson ImmunoResearch Inc.), and visualized with enhanced chemiluminescence reagents (ECL system; GE Healthcare).

Immunofluorescence and confocal microscopy

Confluent MSC-monolayer growing on fibronectin-coated glass coverslips were cell surface labeled as described[4]. Briefly, cells were gently washed with Ca/Mg-PBS (1 mM $CaCl_2$, 0.5mM $MgCl_2$ in PBS), first at room temperature and then on ice, blocked with immunofluorescence buffer (Ca/Mg-PBS containing 2% FCS), and surface labeled with primary antibodies (Supplementary Table S2) for 30 min at 4°C. Unbound antibodies were removed by three washes with ice-cold immunofluorescence buffer. Afterwards, cells were fixed with 4% PFA for 30 min and quenched with 50 mM NH_4Cl for 10 min. For N-cadherin, nestin and vimentin, PFA–fixed cells were permeabilized with 0.2% saponin prior the immunolabeling. Primary antibodies were detected with an appropriate fluorochrome-conjugated secondary antibody: donkey anti-rat DyLight®549, anti-rabbit DyLight®549,

anti-goat Alexa Fluor®546, anti-mouse DyLight®549 or goat anti-hamster Alexa Fluor®546 (Jackson ImmunoResearch Inc). GM1 was detected by Alexa Fluor®488 conjugated Cholera Toxin Subunit B (Life Technologies). Nuclei were labeled with 4,6-diamidino-2-phenylindole (DAPI; 1 µg/ml, Life Technologies). The samples were mounted in Mowiol 4.88 (Merck). Images were captured using a Leica SP5 upright confocal laser scanning microscope. All images shown were processed with Adobe Photoshop software.

Time-lapse microscopy

Freshly isolated $CD34^+$ human hematopoietic stem and progenitor cells (HSPCs) were seeded on either murine or human MSC monolayer growing on 24 x 60 mm fibronectin-coated glass coverslips attached to a reusable silicone chamber and cultured for one or seven days. During the time-lapse recording, cells were kept in a chamber with 5% CO_2 atmosphere at 37ºC. Serial phase-contrast images were captured at 30-sec intervals for 30 min with an inverted microscope (Leica AF6000 LX; Leica). The manual tracking of the HSPCs was done with ImageJ[5]. The time-lapse recording of MSCs cultured without or with 5 mM EGTA (BDH Chemicals) was performed for 60 min.

Scanning electron microscopy

Samples for the scanning electron microscopy analysis were prepared as described[6]. Briefly, co-cultured cells growing for seven days on fibronectin-coated coverslips were fixed in 2% glutaraldehyde for 1 hour at room temperature and then overnight at 4°C. After being subjected to dehydration in an acetone gradient (25–100%), cells were critical point-dried in a CO_2 system (Critical Point Dryer CPD 030, BAL-TEC GmbH). Samples were then sputter-coated with gold (Sputter Coating Device SCD 050, BAL-TEC GmbH) and examined at 5-kV accelerating voltage in an environmental scanning electron microscope (JSM-7500F, Jeol).

Atomic force microscopy-based single-cell force spectroscopy

To measure specific adhesion force, we seeded MSCs following three different protocols as depicted in Supplementary Fig. S8. First, MSCs were grown on fibronectin-coated 30-mm Petri dish (TPP) until 30-70% confluence (Supplementary Fig. S8a) or confluence (Supplementary Fig. S8b). In the latter situation, the confluent feeder layer was partly scratched with a pipette tip to get an MSC-free area. Alternatively, MSC were cultured on a fibronectin-coated coverslip, which was placed into a 30-mm Petri dishes (TPP) prior atomic force microscopy-analysis (Supplementary Fig. S8c). In all cases, the MSC-free area was used to load a single HSPC to the cantilever. Similar data were obtained with these protocols (data not shown).

Supplementary References

1 Morikawa, S. *et al.* Prospective identification, isolation, and systemic transplantation of multipotent mesenchymal stem cells in murine bone marrow. *J Exp Med* **206**, 2483-2496 (2009).

2 Solchaga, L. A. *et al.* FGF-2 enhances the mitotic and chondrogenic potentials of human adult bone marrow-derived mesenchymal stem cells. *J Cell Physiol* **203**, 398-409 (2005).

3 Corbeil, D., Fargeas, C. A. & Huttner, W. B. Rat prominin, like its mouse and human orthologues, is a pentaspan membrane glycoprotein. *Biochem Biophys Res Commun* **285**, 939-944 (2001).

4 Corbeil, D., Röper, K., Hannah, M. J., Hellwig, A. & Huttner, W. B. Selective localization of the polytopic membrane protein prominin in microvilli of epithelial cells - a combination of apical sorting and retention in plasma membrane protrusions. *J Cell Sci* **112**, 1023-1033 (1999).

5 Rasband, W. S. U.S. (National Institutes of Health, Bethesda, Maryland, USA) http://imagej.nih.gov/ij/. (1997). Date of access: 21/01/2015

6 Freund, D. *et al.* Polarization of human hematopoietic progenitors during contact with multipotent mesenchymal stromal cells: effects on proliferation and clonogenicity. *Stem Cells Dev* **15**, 815-829 (2006).

Supplementary Figures

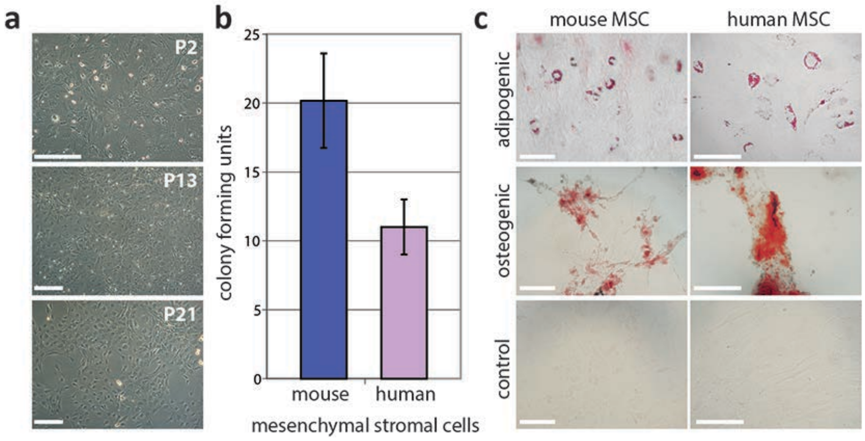

Figure S1: Proliferation, clonogenicity and multipotential differentiation capacity of murine MSCs. (**a**) Plastic-adherent murine (m) MSCs display fibroblastoid morphology. Cells were observed by phase-contrast microscopy at passage (P) 2, 13 and 21. (**b**) CFU-F assays. Murine and human (h) MSCs were seeded (250 cells per dish) at passage 4 and total numbers of colonies were counted after 14 days. Mean and standard deviation are shown (n = 6). (**c**) Differentiation of m and hMSCs. Cells were cultured in adipogenic or osteogenic differentiation medium for 14 and 21 days, respectively, prior to labeling with Oil Red O for the detection of intracellular lipid vesicles (adipogenic differentiation) or Alizarin Red S for calcium-rich deposits (osteogenic differentiation). As controls, undifferentiated cells were labeled with Oil red O (not shown) or Alizarin Red S (control). Data presented were generated from one preparation of m or hMSCs (passage 4), but similar results were obtained with other cell preparations (data not shown). Scale bars: 200 µm.

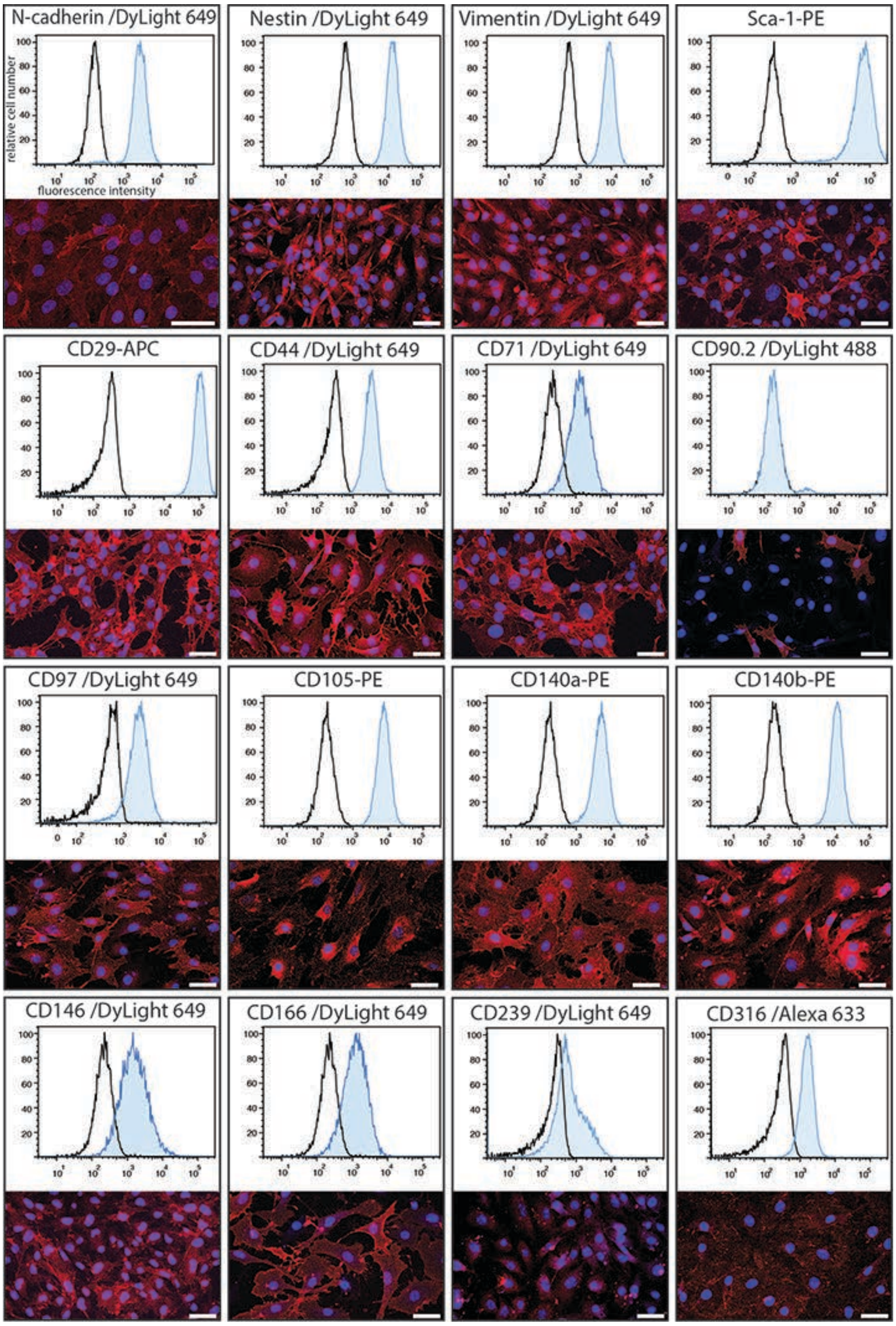

Figure S2: Phenotypic characterization of murine MSCs – Part I. Undifferentiated MSCs were analyzed by flow cytometry (top panels) or confocal microscopy (bottom panels) using a panel of primary antibodies as indicated. For the detection of CD325 (N-cadherin, antibody: 32/N-cadherin clone), nestin and vimentin, PFA-fixed cells were permeabilized with 0.2% saponin prior to immunolabeling. For flow cytometry analysis, fluorochrome-conjugated secondary antibodies were used when primary ones were

unlabeled. The antigen expression (blue) and the appropriate isotype control (black) are shown. For the microscopic analysis, the appropriate fluorochrome-conjugated secondary antibody was used, and nuclei were visualized with DAPI. Data presented were generated from one preparation of mMSCs, but similar results were obtained with two other cell preparations (data not shown) with the notable exception of CD90.2 (see Results). For negative controls, see Supplementary Fig. S3. Scale bars: 50 μm.

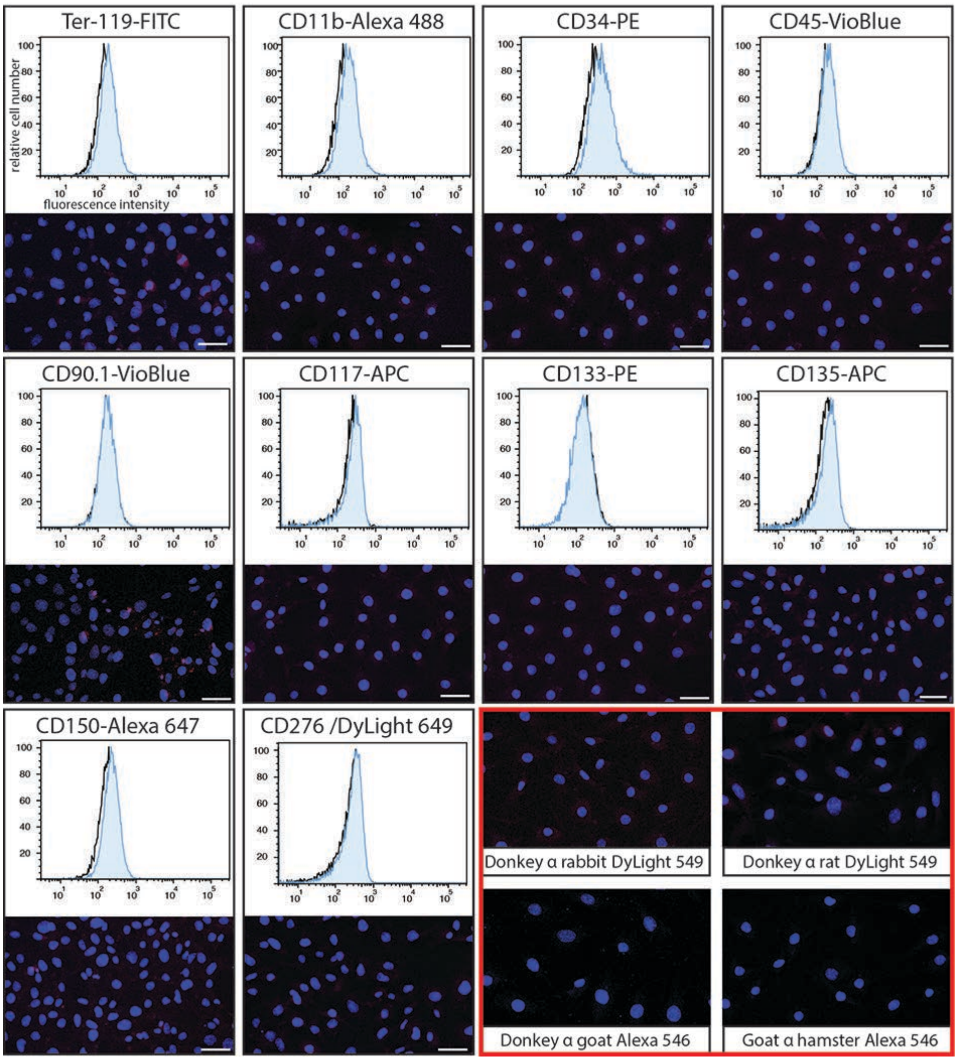

Figure S3: Phenotypic characterization of murine MSCs – Part II. Undifferentiated MSCs were analyzed by flow cytometry (top panels) or confocal microscopy (bottom panels) using a panel of primary antibodies as indicated. For flow cytometry analysis, fluorochrome-conjugated secondary antibodies were used when primary ones were unlabeled. The antigen expression (blue) and the appropriate isotype control (black) are shown. For the microscopic analysis, the appropriate fluorochrome-conjugated secondary antibody was used, and nuclei were visualized with DAPI. Data presented were generated from one preparation of mMSCs, but similar results were obtained with two other cell preparations (data not shown). As negative control (red box), only the secondary antibody as indicated was used. Scale bars: 50 µm.

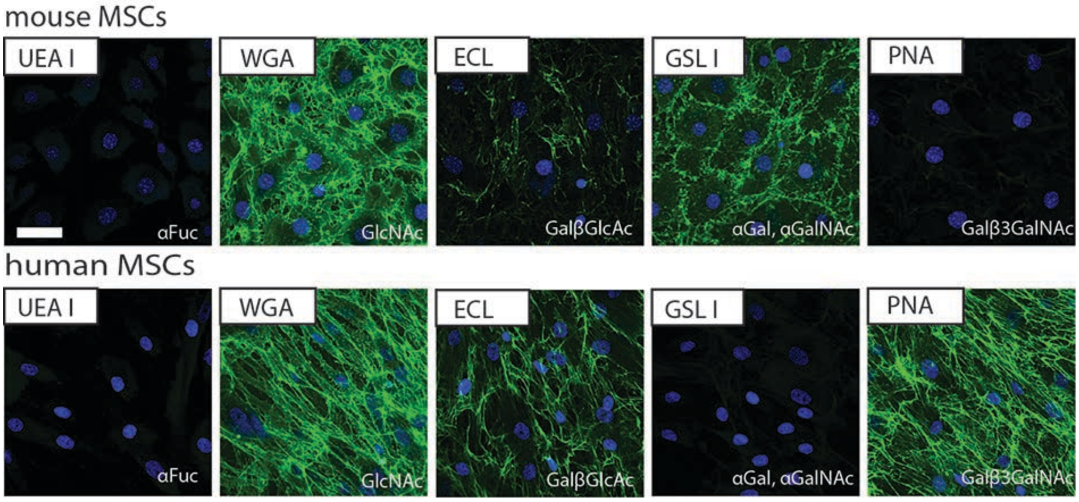

Figure S4: Lectin binding to MSCs. Plastic-adherent murine (m) and human (h) MSCs were cell-surface labeled with distinct fluorescein isothiocyanate-conjugated lectins. Nuclei were visualized with DAPI (blue). A selection of 5 out of 20 used lectins is shown. See Supplementary Table S1 for their abbreviations. Lectin binding group is indicated. Fuc, L-Fucose; Gal, D-Galactose; Glc, D-Glucose; GalNAc, N-Acetylgalactosamine; GlcNAc, N-Acetylglucosamine. Data presented were generated from one preparation of mMSCs, but similar results were obtained with two other cell preparations (data not shown). The data with hMSCs are in agreement with a previous study (see Results). Scale bars: 50 µm.

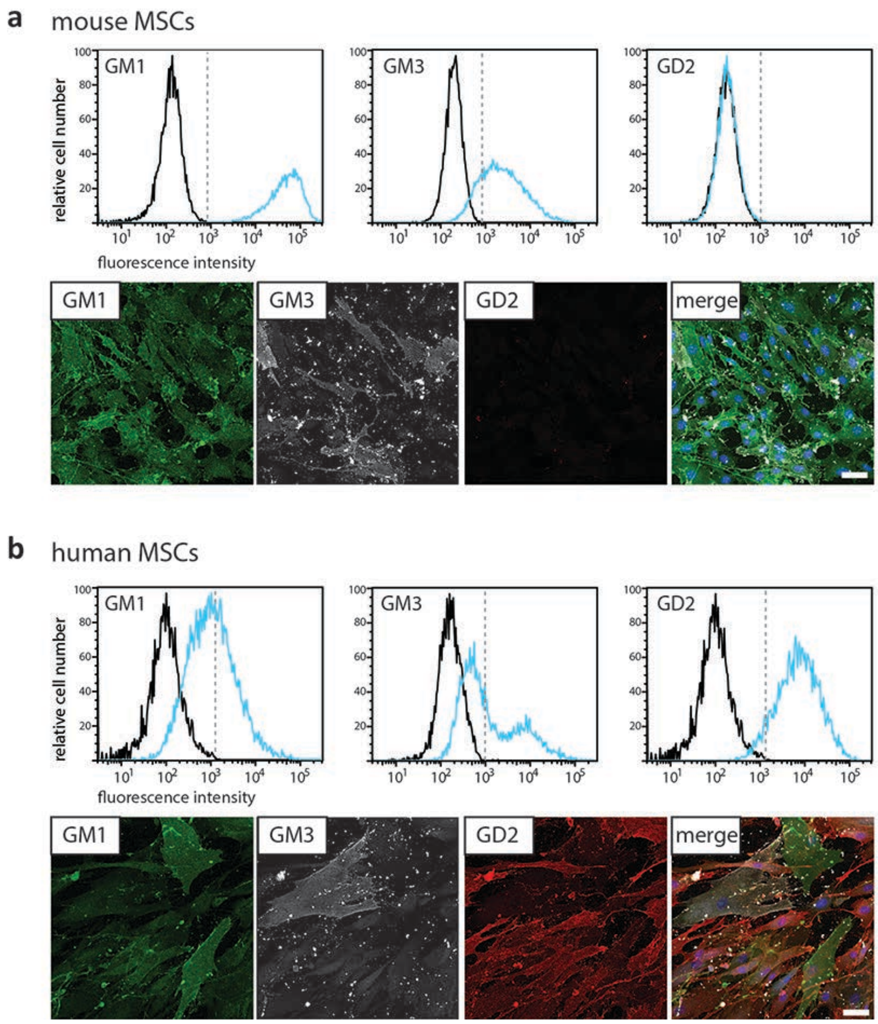

Figure S5: Expression of gangliosides in MSCs. (**a**, **b**) Murine (a) and human (b) MSCs were cell surface-labeled for GM1, GM3 and GD2 and analyzed by flow cytometry (upper panel) and confocal scanning microscopy (lower panel). For flow cytometry analysis, the antigen expression (blue) and the appropriate control (black) are shown. For the microscopic analysis, cells were cell surface triple-labeled for GM1 (green), GM3 (white) and GD2 (red) and counterstained with DAPI for visualization of the nuclei. A composite image of several optical x-y plane sections is shown. Data presented were generated from one preparation of mMSCs, but similar results were obtained with two other cell preparations (data not shown). The data with hMSCs are in agreement with a previous study (see Results). Scale bars: 50 µm.

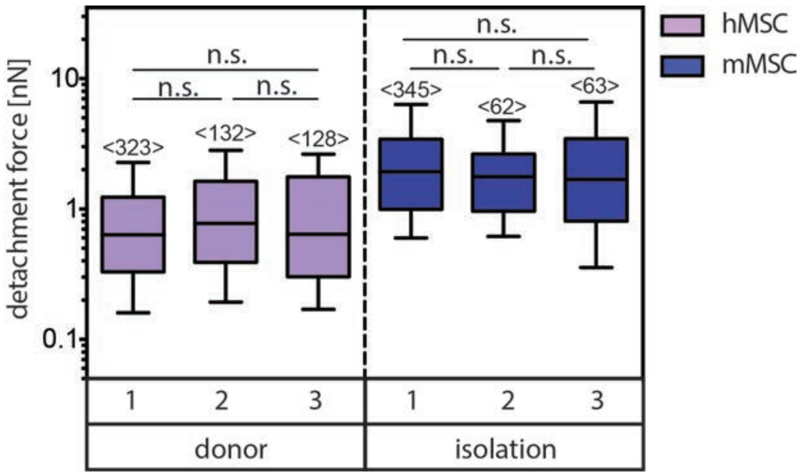

Figure S6: MSCs derived from distinct donors or cell isolations from murine BM do not influence the detachment force of human HSPCs. Box-and-whisker plots show F_D of freshly isolated CD34$^+$ hHSPCs measured by AFM after 60 sec of contact with human (h) and murine (m) MSCs that are derived from three individual donors (in case of hMSCs) or distinct cell isolations (in case of mMSCs). Data presented in figure 4 (i.e. MSCs derived from donor 1 or isolation 1) are indicated for comparison. Box-whisker plots represent half of the data points within the box and 80% within the whiskers. Horizontal lines within the box represent median values. Numbers within bars (<*n*>) show the total number of analyzed F_D curves. The pairwise comparison of data was performed using Mann-Whitney-U-test. N.s., not significant ($p \geq 0.05$).

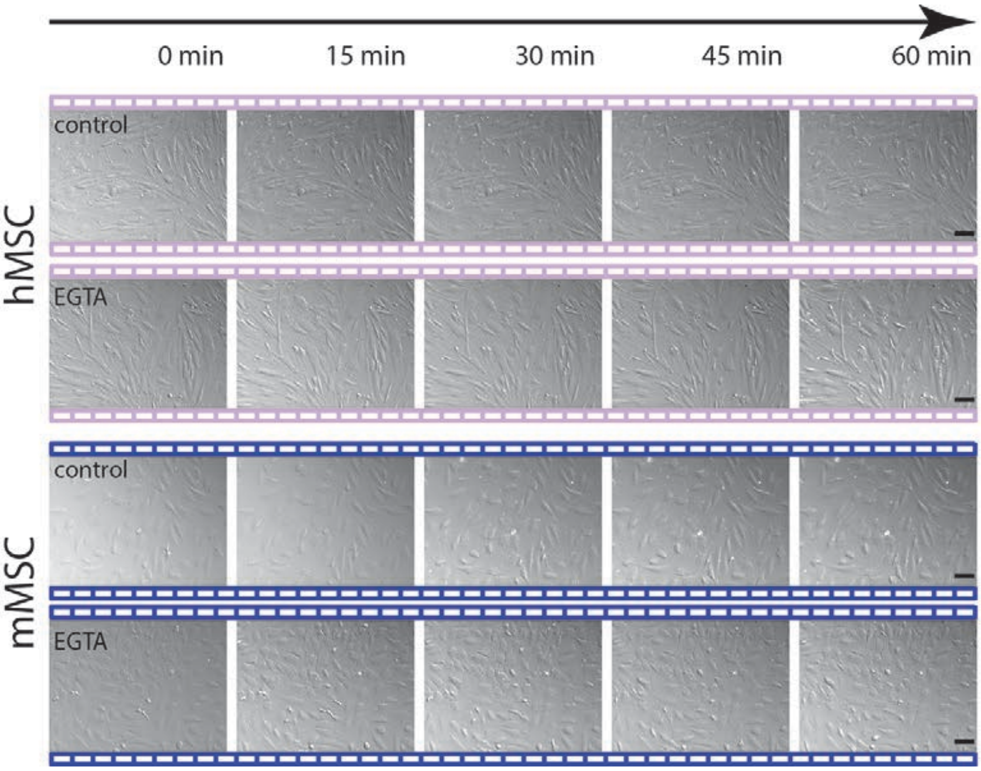

Figure S7: EGTA does not affect the adhesion of MSCs to the substratum and their spindle shaped morphology. Individual frames from a time-lapse microscopy of human (h) and murine (m) MSCs cultured in absence (control) and presence of 5 mM EGTA. A time period of 60 min is shown. Scale bars: 100 µm.

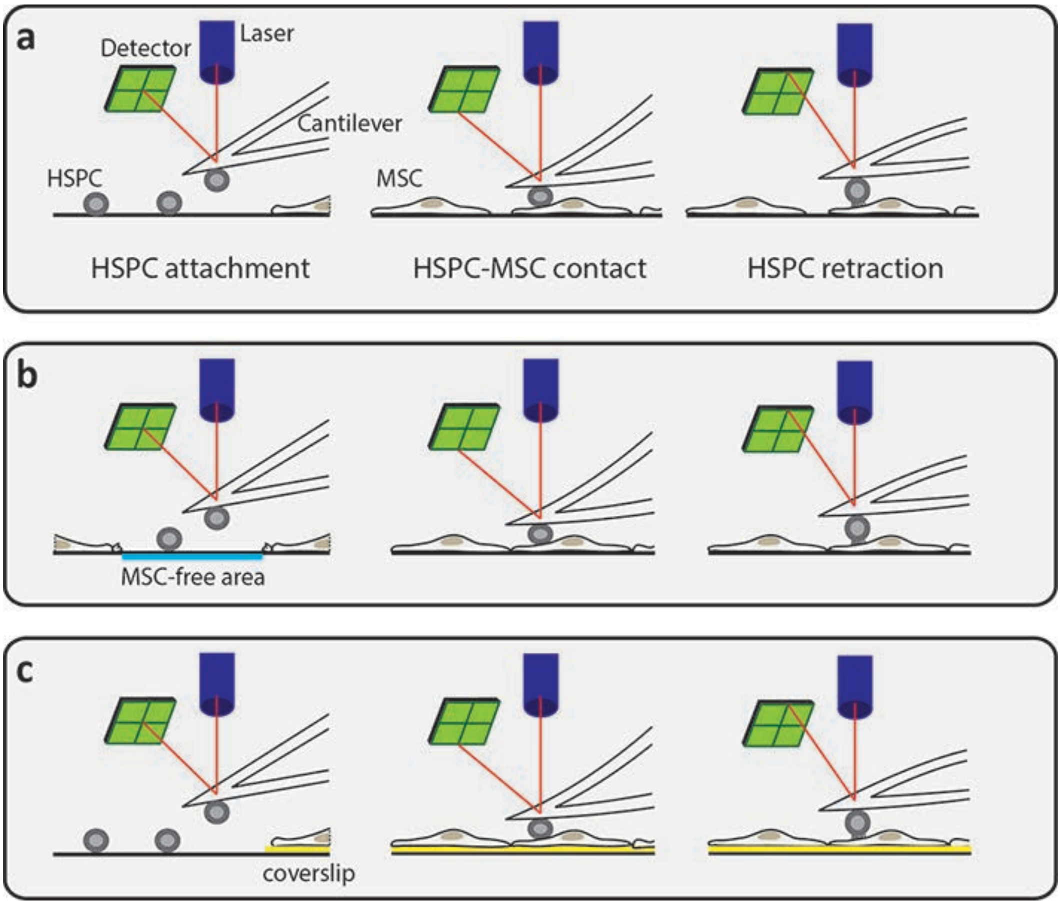

Figure S8: Seeding of MSCs for the AFM analysis. (**a-c**) Three protocols of MSC-seeding were used to analyze the interaction of a single hHSPC with a MSC. MSCs were cultured on fibronectin-coated dishes until 30-70% confluence (protocol in panel a) or confluence (protocol in panel b). In the second procedure, the confluent MSC layer was scratched with a pipette tip to create a cell-free space (cyan line) prior to the seeding of hHSPCs. Alternatively, MSCs were grown to confluence on a fibronectin-coated coverslip (yellow), which was placed into a Petri dish prior AFM-analysis (protocol in panel c). In all protocols, HSPCs were attached to an AFM cantilever in an MSC-free substratum (HSPC attachment) since adhesive interactions between HSPC and MSC can prevent the initial attachment of hematopoietic cells to the cantilever. After establishing a strong connection to the cantilever, the cantilever-bound HSPC was approached at a constant speed onto a MSC until a contact force of 1 nN was reached (HSPC-MSC contact). After a defined

contact time, the cantilever was retracted until the HSPC was completely separated from the MSC (HSPC retraction). During approach and retraction, a F-D curve was recorded.

Supplementary Tables

Table S1. Lectin binding characteristics of murine and human mesenchymal stromal cells

Lectin[1]	Abbreviation	mMSCs	hMSCs
Concanavalin A	ConA	+	+
Dolichos biflorus agglutinin	DBA	–	–
Datura stramonium lectin	DSL	–	–
Erythrina cristagalli lectin	ECL	+	+
Griffonia simplicifolia lectin I	GSL I	+	–
Griffonia simplicifolia lectin II	GSL II	–	–
Jacalin	Jacalin	+	+
Lens culinaris agglutinin	LCA	+	+
Lycopersicon esculentum lectin	LEL	–	–
Phaseolus vulgaris agglutinin–erythrocytes	PHA-E	+	+
Phaseolus vulgaris agglutinin–leucocytes	PHA-L	+	+
Peanut agglutinin	PNA	–	+
Pisum sativum agglutinin	PSA	+	+
Ricinus communis agglutinin I	RCA I	+	+
Sophora japonica agglutinin	SJA	–	–
Solanum tuberosum lectin	STL	+	+
Ulex europaeus agglutinin I	UEA I	–	–
Vicia villosa lectin	VVL	–	–
Wheat germ agglutinin	WGA	+	+
succinylated wheat germ agglutinin	succ. WGA	+	+

[1]All lectins were conjugated to fluorescein isothiocyanate and purchased from Vector Laboratories (Burlingame, CA, USA)

Table S2. Primary antibodies used for immunodetection of mesenchymal stromal cells

Antigen	Fluorochrome-conjugate[1]	Clone	Isotype[2]	Manufacturer[3]	Dilution	
					FC	ICC
CD11b (αM-Integrin)	Alexa Fluor® 488	M1/70.15	r IgG2b	Life Technologies	1:40	1:40
CD29 (β1-Integrin)	APC	HMb1-1	ah IgG	eBioscience	1:100	1:100
CD34	PE	MEC14.7	r IgG2a	Acris	1:20	1:20
CD44 (H-CAM)	–	IM7	r IgG2b	BD Bioscience	1:10	1:50
CD45	VioBlue	30F11.1	r IgG2b	Miltenyi Biotec	1:20	1:100
CD71 (Transferrin receptor)	–	RI7 217.1.4	r IgG2a	Life Technologies	1:50	1:50
CD90.1 (Thy 1.1)	VioBlue	His51	m IgG2a	Miltenyi Biotec	1:20	1:100
CD90.2 (Thy 1.2)	–	53-2.1	r IgG2a	BioLegend	1:200	1:500
CD97	–	M-245	rab IgG	Santa Cruz	1:50	1:50
CD105 (Endoglin)	PE	MJ7/18	r IgG2a	Miltenyi Biotec	1:20	1:100
CD117 (c-kit)	APC	2B8	r IgG2b	eBioscience	1:100	1:100
CD133 (Prominin-1)	PE	13A4	r IgG1	eBioscience	1:20	1:20
CD135 (Flt3)	APC	A2F10.1	r IgG2a	BD Bioscience	1:50	1:50
CD140a (PDGFRα)	PE	APA5	r IgG2a	BioLegend	1:20	1:50
CD140b (PDGFRβ)	PE	APB5	r IgG2a	BioLegend	1:20	1:50
CD146 (LSEC)	–	ME-9F1	r IgG2a	Miltenyi Biotec	1:20	1:100
CD150 (SLAM)	Alexa Fluor® 647	TC15-12F12.2	r IgG2a	BioLegend	1:200	1:200
CD166 (ALCAM)	–	eBioALC48	r IgG2a	eBioscience	1:100	1:100
CD239 (BCAM)	–	H-44	rab IgG	Santa Cruz	1:50	1:50
CD276 (B7-h3)	–	RTAA15	r IgG2a	BioLegend	1:50	1:50
CD316 (IGSF8)	–	P-13	g IgG	Santa Cruz	1:50	1:50
CD325 (N-cadherin)	–	32/N-cadherin	m IgG1	BD Bioscience	1:100	1:100
CD325 (N-cadherin)	–	GC-4	m IgG1	Sigma-Aldrich	1:100	–
GM3	–	GMR6	m IgM	Seikagaku Corp.	1:100	1:100
GD2	–	14.G2a	m IgG2a	BD Bioscience	1:100	1:100
Ter-119	FITC	Ter-119	r IgG2b	BD Bioscience	1:100	1:100
Vimentin	–	V9	m IgG1	Santa Cruz	1:50	1:50

[1]APC, Allophycocyanin; PE, Phycoerythrin; FITC, Fluorescein isothiocyanate
[2]R, rat; ah, american hamster; m, mouse; g, goat; rab, rabbit
[3]Acris (Herford, Germany), BD Bioscience (Heidelberg, Germany), BioLegend (San Diego, CA, USA), eBioscience (San Diego, CA, USA), Life Technologies GmbH (Darmstadt, Germany), Miltenyi Biotec (Bergisch Gladbach, Germany), Seikagaku Corporation (Tokyo, Japan), Santa Cruz Biotechnology Inc. (Santa Cruz, CA, USA)
FC, flow cytometry; ICC, immunocytochemistry

Table S3. Primary antibodies used for immunodetection of hematopoietic cells

Antigen	Fluorochrome-conjugate[1]	Clone	Isotype	Manufacturer[2]	Dilution (FC)
CD45	VioBlue	5B1	mouse IgG2a	Miltenyi Biotec	1:20
CD133/1	PE	AC133	mouse IgG1	Miltenyi Biotec	1:20
CD34	APC	581	mouse IgG1	BD Bioscience	1:10
CD38	FITC	HIT2	mouse IgG1	BD Bioscience	1:10

[1]APC, Allophycocyanin, PE, Phycoerythrin, FITC, Fluorescein isothiocyanate
[2]Miltenyi Biotec (Bergisch Gladbach, Germany); BD Bioscience (Heidelberg, Germany)
FC, flow cytometry
